# Awareness, Knowledge, and Interest about Prebiotics—A Study among Romanian Consumers

**DOI:** 10.3390/ijerph19031208

**Published:** 2022-01-21

**Authors:** Gabriela Precup, Cristina Bianca Pocol, Bernadette-Emőke Teleky, Dan Cristian Vodnar

**Affiliations:** 1 Faculty of Food Science and Technology, University of Agricultural Sciences and Veterinary Medicine, 400372 Cluj-Napoca, Romania; gabriela.precup@usamvcluj.ro (G.P.); bernadette.teleky@usamvcluj.ro (B.-E.T.); 2 Department of Animal Production and Food Safety, University of Agricultural Sciences and Veterinary Medicine, 400372 Cluj-Napoca, Romania; cristina.pocol@usamvcluj.ro; 3 Institute of Life Sciences, University of Agricultural Sciences and Veterinary Medicine, 400372 Cluj-Napoca, Romania

**Keywords:** consumer behavior, prebiotics, evidence, attitude, food, education

## Abstract

The consumer awareness towards healthier diets and the impact of nutrition on health has triggered an increase in the production and commercialization of foods with health claims. The scientific literature classifies these food products as functional foods, with a role in promoting health and preventing diseases, and they had a market share of almost 200 million EUR in 2019. Prebiotics are considered functional foods, referring to substrates that are selectively utilized by host microorganisms conferring a health benefit, as defined by the International Scientific Association for Probiotics and Prebiotics. Several health benefits are associated with the consumption of prebiotics; however, specific requirements must demonstrate the causality between the specific ingredient and the claimed effect. Health claims associated with food products are assessed in the European Union and need to be supported by rigorous scientific evidence before being authorized and permitted on the market. Consumers’ perception of this topic is influenced by the various stakeholders involved. The current work aimed to study the consumers’ perception and interest and to assess the knowledge on the prebiotic concept in Romania. The consumer interest level was quantified by using the web-based data tool Google Trends, and a questionnaire-based investigation was designed. The collected data were analyzed with the help of the SPSS program, and crosstabulation was used to identify the influence of socio-demographic characteristics on diet choice and awareness of prebiotics. A total of 303 persons answered the online applied questionnaire, grouped as young consumers (15–24 years old) and adults (25–64 years old). Even if most responders were familiar with the term of prebiotics (74% of total responders), some results were contradictory regarding their knowledge. The work emphasized the need to carry out educational campaigns and inform consumers on the relationship between certain food ingredients and health outcomes in a clear way and based on a rigorous assessment of the scientific evidence.

## 1. Introduction

In recent years, the shift of the consumer’s behavior towards healthier diets and nutritious foods increased tremendously since researchers linked some food ingredients with potential health benefits [1,2,3,4]. The growing demand triggered an increase in the global market for functional foods, especially for the so-called “prebiotic” compounds and “probiotics” [1,4,5,6]. Recent studies associated the consumption of prebiotics with potential beneficial effects on human and animal health, namely on the gastrointestinal tract (GI), obesity, type 2 diabetes mellitus, irritable bowel syndrome and inflammatory bowel disease, cardiovascular diseases, bones, and neurological disorders, such as anxiety, depression, and cognitive deficiency [2,7,8,9,10,11]. While there is a general consumer perception that prebiotics impart beneficial effects, there is still a lack of knowledge in understanding the definition, while the mechanisms of effect and health attribute still need to be elucidated.

The concept of “prebiotics” was coined in 1995 by Gibson and Roberfroid, referring to a feeding ingredient resistant to digestion, fermentable in the colon ecosystem, and that is able to stimulate the growth and/or the activity of specific microorganisms from the gut, thus being beneficial in the intestinal physiology [1]. The increasing knowledge on the complexity of the gut microbiota and its interaction with the host expanded the concept and definition of prebiotics as a consequence of the advances in microbiome research and the advent of high-throughput sequencing techniques [10]. Therefore, the latest definition was given in 2018 by a panel of experts from the International Scientific Association for Probiotics and Prebiotics (ISAPP) as “a substrate that is selectively utilized by host microorganisms conferring a health benefit” [12].

In this regard, the body of evidence reporting the potential beneficial effects of some nutrients, such as inulin and oligofructose (produced from inulin) and fructooligosaccharides (FOS) synthetically produced from sucrose as well as galactose-containing and xylose-containing oligosaccharides (xylooligosaccharides), began to appear in the 1980s and early 1990s [5,13,14]. Japanese researchers were the first who discovered the value of non-digestible oligosaccharides. They showed that the intake of FOS and galactooligosaccharides (GOS) found in human milk stimulated the growth of intestinal bifidobacteria within the human gut [13,14].

The interplay between prebiotics and probiotics as food or drug triggered the need for specific requirements to be classified as prebiotics as defined by ISAPP, a non-profit organization promoting probiotic and prebiotic science [12], whose definition is as follows: firstly, the ability to be resistant to gastric acidity, enzymes hydrolysis, and gastrointestinal absorption; selectively fermented by intestinal microorganisms; and selectively target and stimulate the growth and activity of gut bacteria [1,12,15]. In-vitro and in-vivo studies are needed to demonstrate a substrate’s health benefit with potential prebiotic effects. In-vitro tests can be used to screen the potential candidates, and then, clinical trials are required to quantify the target bacteria and demonstrate the prebiotic effect. As highlighted by early research, the most common microorganisms shown to “selectively” utilize prebiotics were *Bifidobacterium* and *Lactobacillus* [16,17,18,19]. However, due to the limitations of culture methods, the evidence was insufficient to demonstrate the changes in the gut microbial ecosystem that could utilize the prebiotic substrates. Still, in the past decade, the high-throughput sequencing techniques revealed other autochthonous gut bacteria, including *Faecalibacterium prausnitzii* and *Akkermansia muciniphila,* that increased in abundance as a response to prebiotic intake [9,20,21,22].

Furthermore, prebiotics and probiotics could be combined into “synbiotics” [23], so synergetic effects could be obtained, as the probiotic strain might be stimulated to grow by fermenting the prebiotic [6,24]. As of January 2022, there were 277 registered clinical trials (https://clinicaltrials.gov/ct2/results?term=prebiotics&Search=Apply&recrs=e&age_v=&gndr=&type=&rslt=, accessed on 6 January 2022) worldwide, the majority in Europe, which evaluated the effect of prebiotics (alone or in combination with probiotics) on body weight and obesity metabolic syndrome, type 2 diabetes mellitus, gastrointestinal disorders (diarrhea, constipation, enterocolitis), irritable bowel syndrome and inflammatory bowel disease, and cardiovascular diseases but also on anxiety disorders, arthritis, or allergies [25,26,27,28,29,30,31,32,33]. The associated health outcomes of prebiotics in the GI tract highlighted in the literature were linked to the stimulation of immune system by inhibiting the potential for the growth of pathogenic microorganisms; production of short-chain fatty acids, such as acetate propionate and butyrate; lowering the intestinal pH; and promoting mineral absorption.

However, in the European Union (EU), any health claim on food has to be substantiated by scientific evidence, requiring assessment by the European Food Safety Authority (EFSA) and authorization by the European Commission (EC). Food business operators must follow the provisions of the Regulation (EC) No. 1924/2006 when they want to label or advertise the particular beneficial effects of their products in relation to health and nutrition. To date, the only authorized health claim by EU as defined in Art. 13.5 from the regulation mentioned above refers to chicory inulin, which contributes to normal bowel function by increasing stool frequency at a daily intake of 12 g chicory inulin. Other compounds, such as GOS, XOS, isomalto-oligosaccharide, or sugar cane fiber, were authorized as novel food ingredients by the EC after a positive opinion by EFSA on their safety for human consumption for specific proposed uses [34,35,36,37].

### Prebiotic Ingredients and Sources for Food Applications

The main prebiotic actors from the current body of evidence are the inulin-derived fructans (FOS, inulin, and oligofructose), derived from various crops, or sucrose and GOS, manufactured from lactose, a waste product of the dairy industry. The inulin-derived fructans (FOS) are found in various vegetable species, such as onion, garlic, celery, asparagus, chicory roots, Jerusalem artichokes (topinambur), and yacon potato, but also in some grains and cereals, such as wheat and barley [29,30,38,39,40]. Human milk is another source of oligosaccharides (HMOs), which are structurally similar to GOS and are essential for newborn babies’ metabolic and immunological systems [31]. However, most foods contain only trace levels of these nutrients; therefore, researchers have found alternatives to produce them by synthesis, enzymatic, thermal, or chemical processes [41].

Waste biomass from the agro-food industry, such as corn cobs and cereal by-products (wheat bran, straws, etc.), could represent a novel source for extracting compounds to be used for various food applications [42,43,44]. An example would be xylooligosaccharides (XOS), obtained from the hemicelluloses fraction of those waste materials [31]. Recent studies reported that polyphenols, polyunsaturated fatty acids (PUFAs), proteins, hydrolysates, and peptides might be potential candidates of prebiotic compounds [45].

Prebiotics were utilized in food applications, such as fermented milk/yogurts, breakfast cereals, sports or health drinks, energy bars, sugar-free candy, chewing gum, baked goods, meat products, pet foods, and feed or in food packaging [46,47,48,49,50,51,52,53,54]. However, there is no consensus on a daily intake of prebiotics regarding the dose until now.

**Figure 1:**
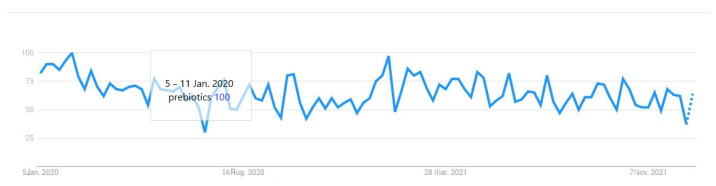
The level of interest in “prebiotics” from December 2019 to December 2021 (https://trends.google.com/, accessed on 6 January 2022).

Finally, since the outbreak of COVID-19, consumers’ interest worldwide in prebiotics had an increasing curve, as observed by simply examining Google search trends on this specific term (Figure 1). It was noticed that the search interest reached the maximum popularity of the term in January 2020 compared to December 2019 and the whole of 2021. Additionally, the body of evidence investigating the role of prebiotics and probiotics in immunity during respiratory infections emerged [55].

Considering the growing interest in these type of compounds, the present work aimed to investigate the current status of prebiotics research in Romania and explore the awareness and knowledge on this topic among Romanian consumers and how socio-demographic characteristics influence the behavior of consumers regarding dietary choices. The work could potentially help the interested stakeholders to address consumer needs and preferences regarding food applications with claimed effects.

## 2. Materials and Methods

### 2.1. Consumer Interest Level for Prebiotics in Romania

To investigate and quantify the level of interest of consumers from Romania on prebiotics, the web-based data tool Google Trends was used, which became a very popular source for extensive data research and was demonstrated useful in assessing and predicting aspects of human behavior [56]. Google Trends offers data on the search volume for specific search terms in a “search volume index” (SVI) from 0–100, representing the relative search volume for a search term indexed against the overall search volume. Data are not given the actual volume and can be classified by category, time, and geographical location (worldwide, country, state, metropolitan region, and city). Peak search activity over a specific period is graded as 100%, and search activity is presented relative to that peak at all other time points. We queried Google trends via the Google Insights for Search (http://google.com/trends, accessed on 6 January 2022) using terminology related to prebiotics in Romanian from May 2018 to December 2021.

### 2.2. Online Survey

#### 2.2.1. Participants

Participants living in Romania and Romanians settled abroad were asked to anonymously complete an online questionnaire about the knowledge, use, and perception of prebiotics, which was published on social media channels and distributed face-to-face among students from the University of Agricultural Sciences and Veterinary Medicine from Cluj-Napoca. Data were collected between May and July 2018. Participants were grouped as young consumers (15–24 years old) and adults (25–64 years old).

#### 2.2.2. Survey Design

As a survey instrument, a brief, two-part questionnaire was designed. It consisted of 10 questions, with both close-ended and open-ended questions. The questionnaire’s application duration ranged between 3 to 5 min, and participants answered the Romanian version of the questionnaire.

#### 2.2.3. Questionnaire Instrument

The questionnaire consisted of close-ended questions and included rating-scale questions (semantic differential), multiple-choice, dichotomous, and open-ended questions. Demographical information was required in the first part, such as gender, location, and education level [57]. The second part contained questions about the type of diet (mixed, semi-vegetarian, ovo-lacto-vegetarian, lacto-vegetarian, total vegetarian, or vegan) and consumption frequency of certain foods or food supplements, respectively, as follows: whole grains (e.g., wheat, barley, oats), wild rice, quinoa, soy, seeds (flax and pumpkin seeds), tomatoes, onions, leeks, garlic, asparagus, artichokes, ginger, radishes, carrots, bananas, berries, apples, dark chocolate, honey, and yogurt. The frequency of consumption of the foods mentioned above was investigated using a measurement scale, which included every day/almost daily, at least once a week, at least once a month, 1–2 times a year, never, or I do not know. Another question assessed their knowledge of the composition of the food mentioned above, which contained non-digestible food ingredients (dietary fiber) classified as prebiotics by the literature.

The respondents were further asked if they were aware of the concept of prebiotics, how they would appreciate their understanding of the concept from a scale from 1 to 10 (where 1 meant they did not know anything about it, and 10 meant they knew a great deal of information), and finally, what was the first thing that came into their mind when hearing this term. To assess their knowledge on the potential beneficial effects associated with prebiotic consumption, the respondents had a multiple choice question with the following options: “improve digestion”, ”support the immune system”, “contribute to a better absorption of nutrients”, ”detoxify the body”, ”contribute to better stress management”, or the option “I do not know/I do not answer.” Finally, to assess the consumers’ perception of the stakeholders responsible for carrying the educational campaigns for informing about the potential beneficial effects, respondents could choose between the family doctor, nutritionist/dietitian, pharmacist, researchers, mass media, family, school, or to mention other means of information.

#### 2.2.4. Statistical Analysis

Data analysis was carried out using Statistical Package for the Social Sciences (SPSS) software program (SPSS Inc., Chicago, IL, USA). Descriptive statistics and crosstabulation were used to identify the influence of socio-demographic characteristics, such as gender, age, and education, on diet choice, prebiotics’ perception, and knowledge. The data are reported as the mean ± standard deviation (SD) [58,59], and differences between means at the 5% level were considered statistically significant.

## 3. Results

### 3.1. Consumer Interest Level

**Figure 2:**
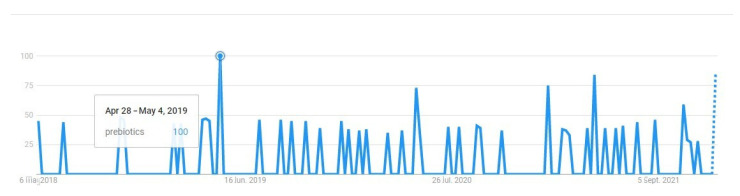
The level of interest in “prebiotics” from May 2018 to September 2021 (https://trends.google.com/, accessed on 6 January 2022).

The search trends for the term “prebiotic” in Romania, analyzed from May 2018 when the questionnaire was applied up to December 2021, had an interesting evolution over the four years. If in 2018 (May–December), the search volume index (SVI) was below 50, from 2019, it increased, with the highest peak (100) between April and May at a constant trend throughout the year. From January 2021, the trend increased from 0 to 75, which could probably be related to the COVID-19 pandemic. People are becoming more interested in foods that could help them maintain good health and improve immunity (Figure 2) [60,61,62]. However, the number of searches is not provided by the tool.

### 3.2. Demographics

A total of 303 questionnaires were obtained through the online investigation, of which one was excluded due to incomplete data. A part of this subsample was extracted from a study made at a national level on 1506 students, conducted during 2017–2018 [63]. The respondents were grouped as young consumers (15–24 years old) and adults (25–64 years old). The young consumers represented 33% of total respondents, with an average age of 22 years old, while the adults represented almost 67% of total respondents and had an average of 33 years old. Male respondents represented 24.2% of the total participants, while women represented 75.8%. Concerning the geographical location of the Romanian consumers, it was noticed that the majority of the respondents were from the county of Cluj (53%), followed by the capital Bucharest (11%) and the county Bistrita-Nasaud (5.3%). A total of 21% of responders were spread in other counties, and Romanians living in other countries represented 6% of the total participants.

**Figure 3:**
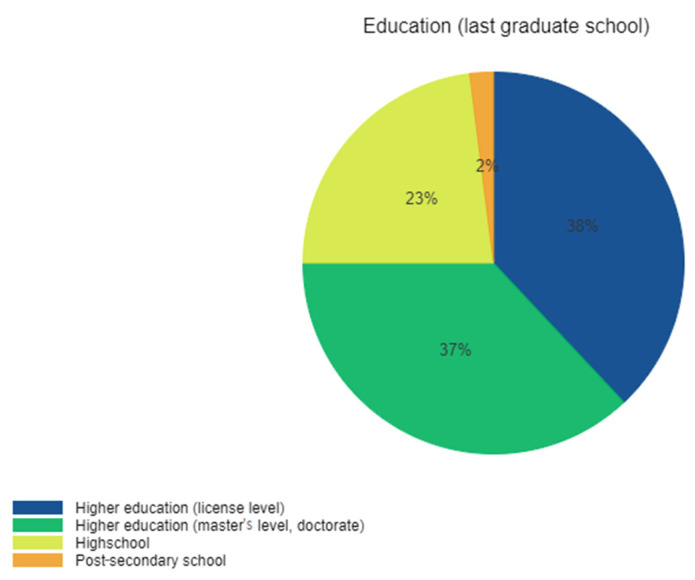
The educational level of the responders.

Concerning the educational level of respondents, the majority (75%) was represented by participants with higher education (bachelor’s degree, master’s, and/or Ph.D. studies) (Figure 3). Demographic characteristics of the study participants are presented in Table 1.

The results of the data analysis showed that the majority of respondents, 84.4%, proclaimed having an omnivorous diet, including both plant and animal foods, of which the majority was represented by women (73%), with a mean age of 30 years old.

**Figure 4:**
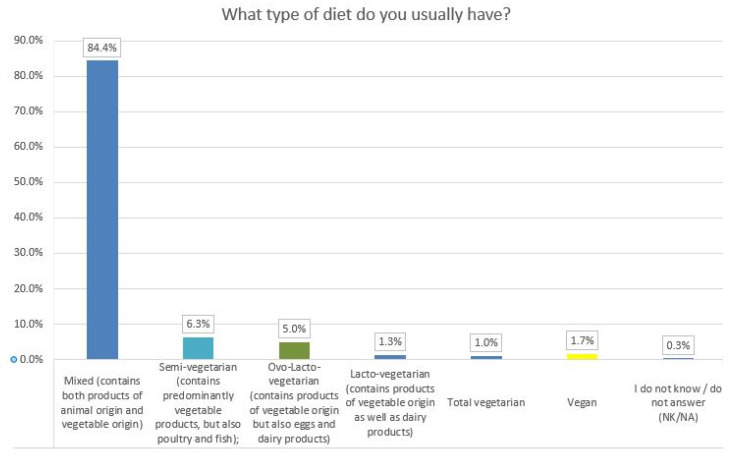
The type of diet reported by participants.

Reduced percentages were recorded in the case of participants who adopted a semi-vegetarian diet (6.3%), ovo-lacto-vegetarian (5%), and lacto-vegetarian (1.3%) (Figure 4). Vegans and vegetarians were poorly represented (1.7% and 1%, respectively). The association between type of diets and respondents’ gender showed that approximately 81% of women and 95% of men had a mixed diet. A percentage of 19% of women with a mean age of 29 years old adopted a vegetarian diet (lacto-, ovo-, semi-vegetarian, vegan diet), while only 5% of men with a mean age of 31 years old were following this dietary lifestyle. Furthermore, the respondents were asked about the frequency (daily, at least once a week, at least once a month, 1–2 times a year, never, or the option I do not know/I do not answer) of consuming some foods that contain inulin-derived fructans (FOS) or other non-digestible compounds.

As observed in Table 2, tomatoes were consumed on a daily basis (around 57 ± 0.9 % of total responses), while onions (45.03 ± 1%), carrots (52.7 ± 0.9%), garlic (51.66 ± 0.9%), whole grains (35.76 ± 1.2%), bananas (48.7 ± 0.9%), apples (40.1 ± 0.9%), yogurt (37.8 ± 1.2%), and honey (24.1 ± 1.2%) are consumed more often once a week rather than daily. At the opposite pole, the food products consumed more rarely, around one to two times a year or never, were represented by artichokes (87.1 ± 0.9%), asparagus (75.9 ± 1%), quinoa (75.6 ± 0.9%), soy (69.7 ± 1%), leeks (69.0 ± 1%), or wild rice (67.3 ± 1.1%). Interestingly, the responders to the survey reported that food supplements were not popular in their diet (84% taking supplements 1–2 times a year or never). However, no association was noticed between the educational level and dietary habits except in the vegetarian and vegan group, where most of the respondents had higher education (bachelor’s, master’s, Ph.D. studies) (82.6 ± 1.1%). In addition, no significant statistical differences were noticed between the frequency of consuming different food products and age groups (young and adults) (*p* > 0.5).

Moreover, more than half of the responders (53 ± 0.53%) were not aware that the food products consumed (Table 2) contained non-digestible compounds that might stimulate the growth of intestinal lactic acid bacteria with potential health outcomes. When considering only respondents with higher education, women were more informed than men (27 ± 0.4% vs. 7 ± 0.1%, *p* < 0.5) on this aspect. However, no significant statistical difference (*p* > 0.5) was observed between women and men with higher education and respondents who graduated only high school and post-secondary school.

**Figure 5:**
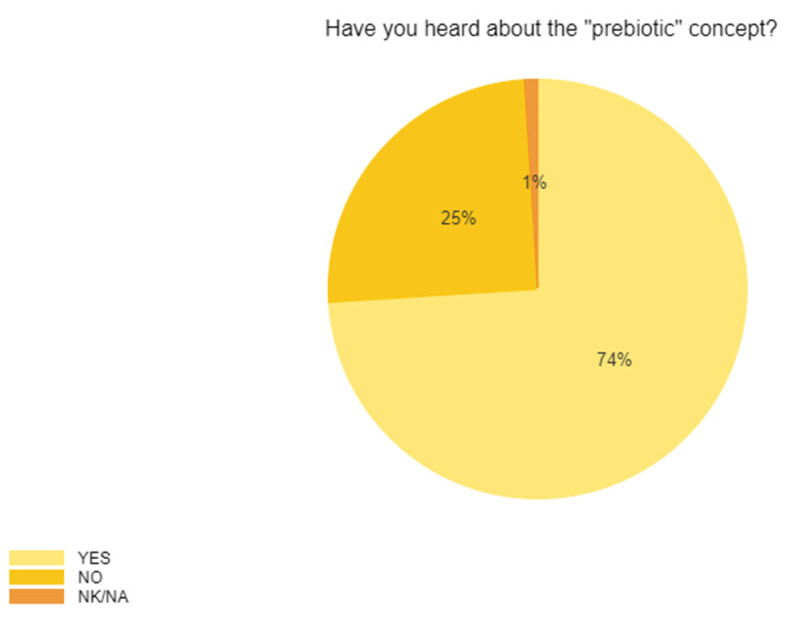
The awareness of the prebiotic concept.

When assessing the awareness of respondents regarding the “prebiotic” concept, a percentage of 74% of the total respondents answered “yes,” whereas 25% had not heard of it (Figure 5). Moreover, when considering only the answers provided by responders from the young group (15–24 years old), which represented 33% of total responders, no significant statistical difference (*p* > 0.5) was observed between responders with higher education (bachelor’s degree, master’s, or Ph.D.) and those with high school and post-secondary school when assessing their awareness to the concept of “prebiotics.” The adult group was represented by responders with a mean age of 30 years old, who were highly educated, and who knew about prebiotics in high number (more than 70 ± 3%). Since women were represented in higher percentages than men in this survey, no comparison was made between these groups.

**Figure 6:**
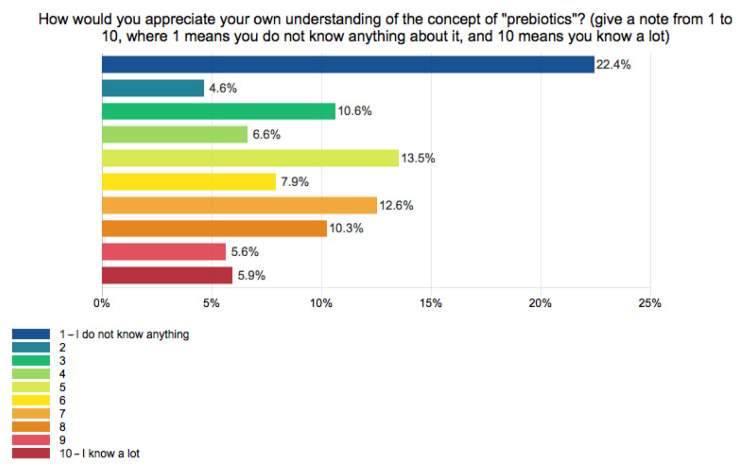
The self-assessment of prebiotic concept knowledge.

When asked to assess their understanding about “prebiotics” on a scale from 1 to 10, 22.4% of responders stated that they had no knowledge, while almost 6% had a great deal of knowledge. Moreover, we compared the responses of those who stated they heard about prebiotics with the self-assessment responses on this concept. We observed that 40 ± 1% of those who were aware of it stated that they also understood the meaning (points from 6 to 10 on the rating scale), while 24 ± 0.5% had not heard. They had little or no knowledge (points from 1 to 4 on the rating scale), and 20 ± 0.5% knew about prebiotics, but they had little or no information. Interestingly, 1% of the responders stated that they had not heard the term “prebiotics” but had knowledge of it (points from 6 to 10 on the rating scale). In addition, some of the first words that would come in their mind when thinking about prebiotics were from different categories related to foods or health outcomes, including “functional foods”, “garlic”, “wheat bran”, “yogurt”, “onion”, “food supplements”, “fibers”, “digestion” “microbiota”, “probiotics”, “prevention”, or “immunity” (Figure 6).

Furthermore, 29.1% of respondents considered that the beneficial effects of consuming foods that contain compounds that might have prebiotic effects were improved digestion, followed by the claims of supporting the immune system (21.3%), contributing to better absorption of nutrients (16.8%), or detoxifying properties (15.6%). Finally, the responders stated that they would consume foods containing potential prebiotic compounds more often if a better awareness existed (84.5% of total respondents).

**Figure 7:**
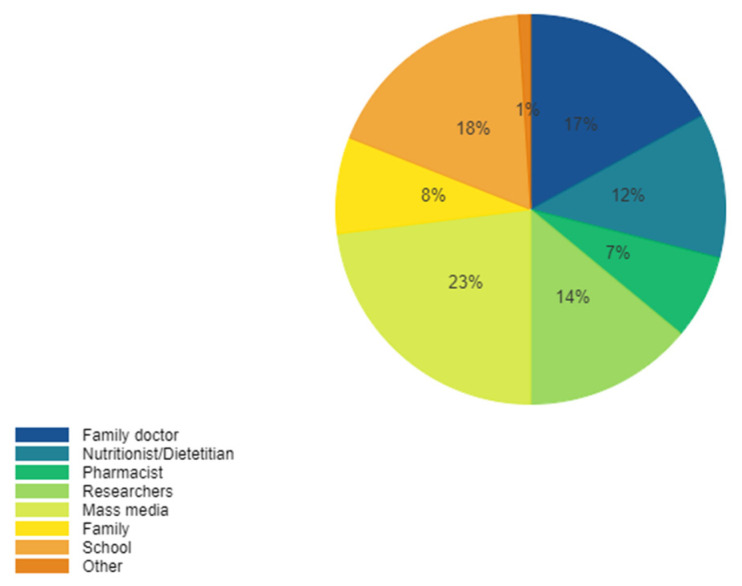
Perception on the stakeholders responsible for consumer’s information on prebiotics.

Finally, regarding the perception on the stakeholders responsible for consumers’ information on prebiotics, the percentages were various: 36%, considered that healthcare providers (family doctors, pharmacists, nutritionists, and dietitians) should be responsible of communicating, while 23% of participants would prefer to have this information from mass-media,18 % thought that the professors in schools should inform on such aspects, whereas 14% chose researchers and 8% their own families. (Figure 7).

### 3.3. Research Status on Prebiotics in Romania

**Figure 8:**
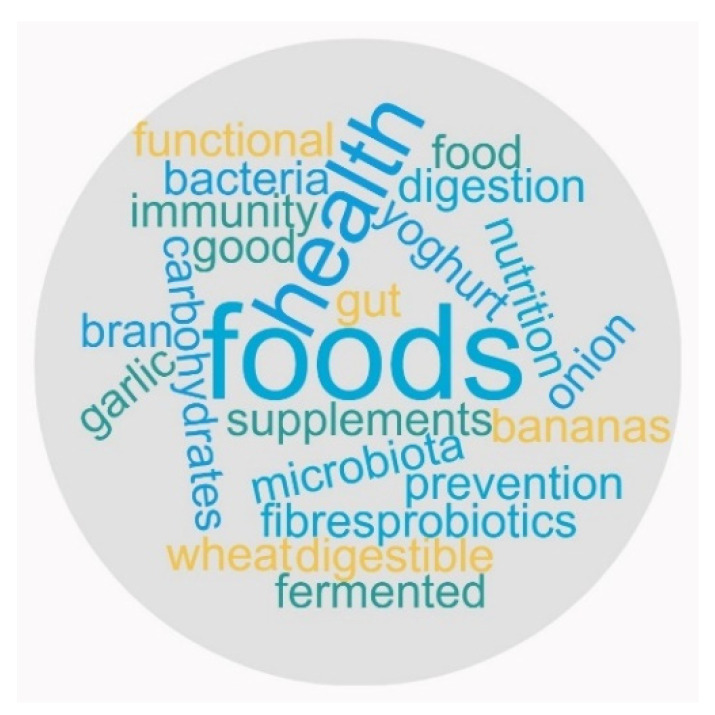
Terms associated with the concept of “prebiotics” by survey responders.

In Romania, inulin was the most studied with respect to its prebiotic potential [40,46,47,64]. It has been used as an ingredient for obtaining functional foods, such as yogurt [46], bread [47], soy milk [48], and vegetable juices [49] (Figure 8). Prebiotic compounds have also been used as feed supplements for bees [50], pigs [51], or chickens [52]. Inulin and pectin were also used to encapsulate probiotic bacteria to test their behavior under gastrointestinal conditions [53,54,65].

Nevertheless, candidate or emerging prebiotics require additional evidence in humans before they can be fully established as prebiotic compounds [41].

## 4. Discussion

About 60% of the functional foods market nowadays focuses on digestive health, with prebiotics and probiotics probably occupying a high market share, as reported by the International Life Sciences Institute (ILSI) [12]. As a result, the prebiotic functional food market has emerged into a multi-million Euro industry, forecasted to register a CAGR of 12.2% until 2024, fueled by the rising health awareness and shift in the consumer lifestyle. Moreover, as a result of continuing development and modernization in the direction of a thriving society, individuals are more inclined towards the functional food sector [66].

However, as reported by recent surveys, the general public’s knowledge of prebiotics is limited [67,68,69,70]. In the USA, a survey from 2015 on 200 U.S. adults (patients at an urban hospital) reported that only 11% were familiar with the term “prebiotics.” Only 7% identified the correct definition among four other choices. Moreover, the most-consumed food products with potential prebiotic compounds were yogurts (72%) and cereals/granola bars (55%). One of the reasons for consumption was the health-related benefits (“digestion or gut health”). Still, the most common reason was driven by curiosity of tasting or trying such foods (“to taste or try”) [69]. The use of these types of ingredients in supplements is constantly increasing, reaching a frequency of 4.5% for pro-, 2.4% for pre-, and 1.1% for synbiotic products. Among consumers, the leading users were older people with higher earnings and educational levels [71,72].

Meanwhile, in Australia, almost 60% [73] and in New Zealand, 25.4% [74] of the interviewed adults had in the past or presently consumed probiotics. In both cases, the highest probiotic preponderance was generally observed among females, especially those with a higher education level. Conflicting results were found regarding the term prebiotic; although 42.8% of the persons who consumed probiotics knew this term, between non-consumers, only 24.7% were familiar with it [73]. In general, adults who pursue healthier ways of life and have enhanced knowledge of gut health and the advantages of pre-, pro-, and synbiotic consumption are more likely to consume these ingredients [75,76].

Another study conducted in India on college students highlighted that the primary source of information for them was from TV advertisements or social media. Still, the term prebiotics was not familiar among the same students except for a few of them [77]. Another survey targeting the perception and attitudes towards functional foods of young students from Turkey reported that more than half (59.7%) of the respondents were not familiar with the functional food concept, and half of the participants might buy functional foods in the future if they would be more informed [67].

In Europe, a study conducted on 105 Slovak students to assess the knowledge on functional foods concluded that only a third of consumers were familiar with the concept, highlighting the need to raise awareness [78]. In Romania, a study conducted in 2016 on 265 consumers to evaluate the knowledge and attitude towards functional foods showed that consumers with high income were more interested in functional foods even if their understanding was not better than low-income consumers. In addition, the same study highlighted a high level of confusion regarding the definition of these types of foods [79].

Our work also emphasized that there is limited general knowledge on the prebiotic concept and that there is a need for better information that should be provided by healthcare providers regarding the association between food consumption and health outcomes. Even if most of the responders were familiar with the term (74% of total responders), when assessing their knowledge, the results were contradictory (40% understood the term, 24% were not familiar and had little or no knowledge, and 20% were familiar but they had little or no information). Surprisingly, even healthcare providers have poor understanding of the prebiotic concept. For example, only 22% out of 256 registered dietitians, nurses, nurse practitioners, physicians, pharmacists, and physician assistants from a medical center in Chicago (USA) have heard of prebiotics. However, more than 83% would recommend prebiotics if proven by literature [70]. Confusion about the differences between probiotics and prebiotics was also common between healthcare providers in another study [80]. The scarce data from the scientific community on prebiotics could be responsible for the lack of knowledge of healthcare providers and highlights the importance of education in order to provide recommendations to patients.

The accelerated interest regarding the positive health effects given by the gut microbiota increased the scientific community’s awareness and the public’s attention regarding the consumption of prebiotic, probiotic, and synbiotic products [81,82,83]. A healthy gut relies on a balanced diet and healthy lifestyle [84] given by high fruit, vegetable, and fiber consumption; limited alcohol; and physical activities [77,79]. In addition, the current COVID-19 pandemic has highlighted the importance of a healthy gut microbiome and its attenuating effect on different respiratory infections [85,86].

## 5. Conclusions

Our study emphasized that the population sample of Romanian consumers representing the young and adult population, mostly women (76%) and highly educated (74% completed or had ongoing bachelor’s degree, master’s, and/or Ph.D. studies), were in general familiar with the concept of prebiotics and some potential health outcomes shown by the scientific literature. However, we noticed scattered confusion among different groups regarding the concept’s meaning. This could be due to the lack of a common consensus and terminology regarding this topic among the involved stakeholders, which leads to misinformation and confusion among consumers.

Future avenues of research should struggle more to confirm the causality between the health effects of emerging prebiotics and microbiota-mediated mechanisms so that healthcare providers can develop evidence-based recommendations. It is also of utmost importance to mention that food business operators who intend to commercialize food products with health claims in the EU should comply with the provisions of the Regulation (EC) No 1924/2006 when they want to label or advertise the particular beneficial effects of their products in relation to health and nutrition. Non-compliance with the Regulation will result in an unauthorized health claim when the claimed effect cannot be substantiated based on the scientific evidence.

## Figures and Tables

**Table 1:** Demographic characteristics of study participants.

Demographics	n	%
Young consumers (15–24 years old)	101	33.4
Adults (25–64 years old)	201	66.6
Gender		
Male	73	24
Female	229	76
**Geographical location (counties/abroad)**		
Alba	4	1.3
Arad	3	1
Arges	1	0.3
Bacau	1	0.3
Bihor	7	2.3
Bistrita-Nasaud	16	5.3
Botosani	1	0.3
Brasov	3	1
Bucuresti	33	11
Buzau	1	0.3
Caras-Severin	1	0.3
Cluj	160	53
Suceava	4	1.3
Galati	5	1.7
Harghita	1	0.3
Hunedoara	2	0.7
Iasi	6	2
Maramures	7	2.3
Mures	3	1
Neamt	2	0.7
Sibiu	3	1
Ialomita	2	0.7
Salaj	3	1
Buzau	1	0.3
Satu-Mare	4	1.3
Timis	7	2.3
Dambovita	1	0.3
Vaslui	1	0.3
Vrancea	1	0.3
**Abroad**		
France	3	1
Germany	5	1.7
Spain	6	2
United Kingdom (UK)	1	0.3
Ukraine	1	0.3
Moldova	2	0.7

**Table 2:** Frequency of consumption of certain food products.

Food Products	Responses (%)		
	Every Day, Almost Daily	At Least Once a Week	1–2 Times a Year or Never
Tomatoes	56.8 ± 0.9 ^a^	32.78 ± 0.9 ^b^	2.65 ± 0.9 ^d^
Onion	38.9 ± 1 ^b^	45.03 ± 1 ^a^	3.9 ± 1 ^d^
Bananas	28.1 ± 0.8 ^b, c^	48.7 ± 0.9 ^a^	2.98 ± 0.9 ^d^
Carrots	23.8 ± 0.8 ^c^	52.7 ± 0.9 ^a^	2.32 ± 0.9 ^d^
Honey	22.8 ± 1.2 ^c^	24.1 ± 1.2 ^c^	19.5 ± 1.2 ^c^
Apples	20.1 ± 0.9 ^c^	40.1 ± 0.9 ^a,b^	8.6 ± 0.9 ^d^
Whole grains	20.86 ± 1.2 ^c^	35.76 ± 1.2 ^b^	0.2 ± 1.2 ^d^
Garlic	13.25 ± 0.8 ^c,d^	51.66 ± 0.9 ^a^	7.2 ± 0.9 ^d^
Berries	12.6 ± 1 ^c, d^	30.79 ± 1 ^b^	19.2 ± 0.3 ^c^
Artichokes	0.6 ± 0.8 ^d^	1.3 ± 0.8 ^e^	87.1 ± 0.9 ^a^
Asparagus	1.9 ± 1 ^d^	4 ± 1 ^e^	75.9 ± 1 ^a^
Wild rice	0	8.2 ± 1 ^d^	67.3 ± 1.1 ^b^
Quinoa	0	4.5 ± 0.9 ^e^	75.6 ± 0.9 ^a^
Soy	0	5.3 ± 1 ^e^	69.7 ± 1 ^b^
Leeks	2.3 ± 1 ^d^	7.2 ± 1 ^d, e^	69.0 ± 1 ^b^
Chia seeds	5.3 ± 1.2 ^d^	10.6 ± 1.2 ^d^	62.7 ± 1.3 ^b^
Yogurt	20.5 ± 1.2 ^c^	37.8 ± 1.2 ^b^	13.2 ± 1.2 ^c,d^
Food supplements	0.05 ± 1.2 ^d^	0	84.2 ± 1.2 ^a^

Results are displayed as mean values ± SD, g/L, *n* = 3; in every column, the significant differences (*p* < 0.05) are displayed with different superscript letters (a–e) between the types of food products. Statistical Package for the Social Sciences (SPSS) software program (SPSS Inc., Chicago, IL, USA).

## Data Availability

The data presented in this study are available within the article. Other data that support the findings of this study are available upon request from the corresponding authors.

## Disclaimer

The author Gabriela Precup is currently employed as Scientific Assistant by Randstad (c/o EFSA)’] and was employed as a trainee at the European Food Safety Authority (EFSA) between 1 November 2019 and 30 April 2021 in the Nutrition Unit that provides scientific and administrative support to the Panel on Nutrition, Novel Foods and Food Allergens in the area “Safety Assessment of Novel Foods”. However, the present article is published under the sole responsibility of the authors Gabriela Precup, Cristina Bianca Pocol, Bernadette-Emőke Teleky and Dan Cristian Vodnar and may not be considered as an EFSA scientific output. The positions and opinions presented in this article are those of the author/s alone and are not intended to represent the views/any official position or scientific works of EFSA. To know about the views or scientific outputs of EFSA, please consult its website under http://efsa.europa.eu (accessed on 16 December 2021).
