# Correction: Precup et al. Awareness, Knowledge, and Interest about Prebiotics—A Study among Romanian Consumers. *Int. J. Environ. Res. Public Health* 2022, *19*, 1208

**DOI:** 10.3390/ijerph20095645

**Published:** 2023-04-26

**Authors:** Gabriela Precup, Cristina Bianca Pocol, Bernadette-Emőke Teleky, Dan Cristian Vodnar

**Affiliations:** 1 Faculty of Food Science and Technology, University of Agricultural Sciences and Veterinary Medicine, 400372 Cluj-Napoca, Romania; gabriela.precup@usamvcluj.ro (G.P.); bernadette.teleky@usamvcluj.ro (B.-E.T.); 2 Department of Animal Production and Food Safety, University of Agricultural Sciences and Veterinary Medicine, 400372 Cluj-Napoca, Romania; cristina.pocol@usamvcluj.ro; 3 Institute of Life Sciences, University of Agricultural Sciences and Veterinary Medicine, 400372 Cluj-Napoca, Romania

## Text Correction

There was an error in the original publication [1]. ** Other compounds, such as GOS, XOS, isomalto-oligosaccharide, or sugar cane fiber, were authorized as novel food ingredients by the EC after a positive opinion by EFSA on their safety for human consumption for specific proposed uses [34–37] as prebiotic ingredients, sources, and for food applications.**

A correction has been made to **Introduction**, **Subtitle in Section 1**:

**Other compounds, such as GOS, XOS, isomalto-oligosaccharide, or sugar cane fiber, were authorized as novel food ingredients by the EC after a positive opinion by EFSA on their safety for human consumption for specific proposed uses [34–37].**

**Subtitle: Prebiotic Ingredients and Sources for Food Applications**

The authors apologize for any inconvenience caused and state that the scientific conclusions are unaffected. This correction was approved by the Academic Editor. The original publication has also been updated.
